# Adherence to the Chinese Food Pagoda in the High-Risk Population of Non-communicable Diseases Aged 35–59 in Central China

**DOI:** 10.3389/fnut.2022.781963

**Published:** 2022-03-04

**Authors:** Qingqing Jiang, Qiqi You, Yiling Lou, Shiqi Wang, Shiyi Cao

**Affiliations:** School of Public Health, Tongji Medical College, Huazhong University of Science and Technology, Wuhan, China

**Keywords:** non-communicable diseases, dietary pattern, Chinese Food Pagoda, adherence, high-risk population

## Abstract

**Objectives:**

A healthy dietary habit is essential for preventing non-communicable diseases (NCDs). We aimed to assess the adherence to the Chinese Food Pagoda (CFP) proposed in the Chinese Dietary Guidelines 2016 in the high-risk population of NCDs in central China.

**Methods:**

A cross-sectional study was conducted in two large enterprises (totally 3,016 employees) from October to December 2019 in Hubei Province (central China). The high-risk population of NCDs was identified by physical examination, laboratory test and face-to-face questionnaire survey according to the *National Norms for Prevention and Control of Non-communicable Diseases* issued by the Chinese government. We assessed the deviation of real diet from the CFP recommended diet in the high-risk population of NCDs.

**Results:**

A total of 821 participants aged 35–59 years old with at least one high-risk factor of NCDs were enrolled in our study. Of them, 53.8% were daily smokers, 49.6% had elevated blood cholesterol, 31.4% were centrally obese, 23.3% had high normal blood pressure, and 3.5% had impaired fasting glucose. Significant disparity was detected in the high-risk population of NCDs between real food consumption and the CFP's recommendation (*P* < 0.05), such as the deficient intake of nuts and milk and dairy products, and the over-consumption of cereals, meat and poultry, oil, and salt. Participants with impaired fasting glucose had the highest intake of cereals and vegetables on average. Participants with central obesity were more likely to consume meat and poultry (*P* < 0.05). The lowest average intake of eggs and the highest average intake of milk and dairy products were found in participants with high blood cholesterol (*P* < 0.05). The daily smokers were more likely to consume beans and nuts (*P* < 0.05). The lowest average intake of fruits and the highest average intake of tubers were found in participants with high normal blood pressure (*P* < 0.05).

**Conclusion:**

Adherence to CFP in the high-risk population of NCDs appeared to be challenging. It is necessary to adopt dietary education campaign focusing on the high-risk population of NCDs to prevent or delay the occurrence of NCDs.

## Introduction

Non-communicable diseases (NCDs) are characterized as chronic and slow-progressing diseases (1), which were collectively responsible for 71% of all deaths worldwide (2). The epidemic of NCDs poses devastating health consequences to individuals, families and communities, and threatens to overwhelm health systems. A healthy diet with a wide variety of foods can provide a range of nutrients to the body, which play an important role in reducing the occurrence of NCDs (3). However, modern lifestyles have led to dietary changes characterized by insufficient fiber intake and increased consumption of processed and ultra-processed foods, which are associated with the increasing risk of NCDs. There is obviously sufficient evidence indicating the association of increased incidence of type 2 diabetes with higher intake of meat and lower intake of cereal fiber. Excessive sodium intake was found to increase the risk of cardiovascular and renal diseases (4), and vitamin D deficiency was associated with a number of non-skeletal disorders, such as heart disease and hypertension (5). Furthermore, many studies pointed out that under-nutrition or over-nutrition were significantly associated with obesity and certain types of cancers (6–9). To achieve the global goal of reducing the burden of NCDs, one of the most important ways is to limit unhealthy lifestyle choices and develop a healthy diet.

**Figure 1:**
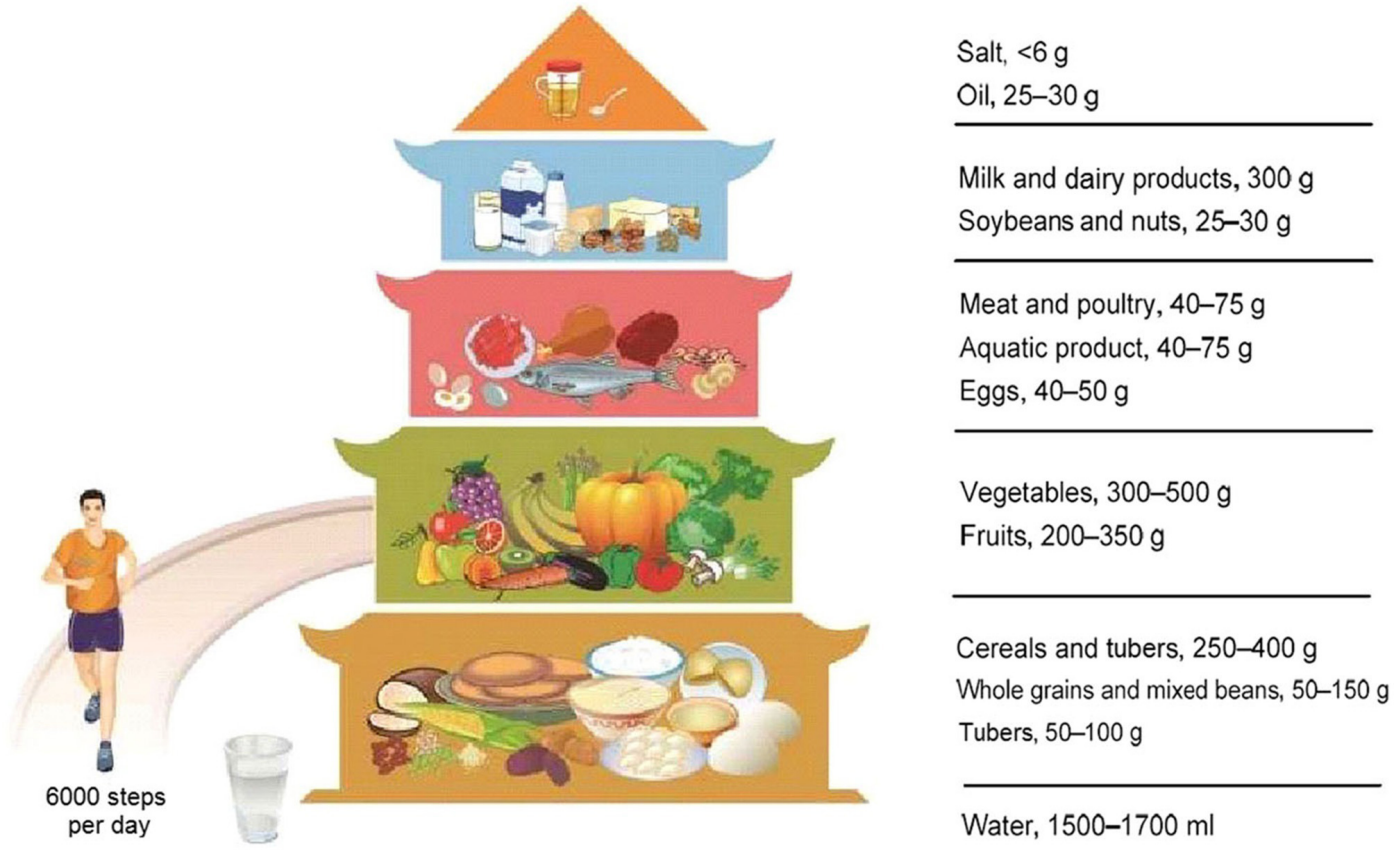
Food Guide Pagoda for Chinese residents.

Fueled by rapid urbanization and changes in lifestyle, China has experienced a dramatic shift from traditional dietary patterns to the diet dominated by high-fat and high-calorie foods (10, 11). The rapid dietary change had led to growing concern on the coexistence of under-nutrition and over-nutrition, which was believed to contribute to the increasing prevalence of NCDs (12, 13). The Chinese government proclaimed a new version of dietary guidelines for Chinese residents in the form of the Food Pagoda 2016 (Figure 1), which recommends a relatively ideal dietary pattern to improve the general nutrition of Chinese residents. Previous studies had reported the deviation of Chinese adults' diet from the Chinese Food Pagoda (CFP) and its association with adiposity (14), cardiovascular disease (15), colorectal cancer (16), breast cancer (17), functional impairments (18), and mortality (15). However, there is limited evidence of adherence to CFP among people with or at high risk of NCDs.

In China, NCDs were responsible for 88.46% of all deaths, and the prevalence was 596.65 per 100,000, making NCDs a critical public health issue (19). There is an urgent need to ascertain dietary patterns of people at high risk of NCDs. Uncovering the difference between their real food consumption and the recommended level in CFP could contribute to more effective guidance on peoples' food consumption habits, which has strong policy implications for alleviating the public burden caused by NCDs. We conducted this cross-sectional study in central China to evaluate the dietary patterns in the high-risk population of NCDs using the CFP 2016. It will be valuable for the future research on daily optimal dietary intake of the high-risk populations of NCDs and for the government to put forward reasonable prevention strategies for NCDs.

## Methods

### Definition of the High-Risk Population of NCDs

In 2011, the National Health Commission of the People's Republic of China issued the *National Norms for Prevention and Control of Non-communicable Diseases* (20), stipulating that those who met at least one of the following criteria was defined as people at high risk of NCDs: (1) high normal blood pressure: 130 mmHg ≤ systolic blood pressure (SBP) ≤ 139 mmHg and/or 85 mmHg ≤ diastolic blood pressure (DBP) ≤ 89 mmHg. (2) Smoking daily. (3) Impaired fasting glucose (IFG): 6.1 mmol/L ≤ fasting plasma glucose (FPG) < 7.0 mmol/L. (4) Elevated blood cholesterol: 5.2 ≤ total cholesterol (TC) < 6.2 mmol/L. (5) Central obesity: waist circumference ≥ 90 cm for men and ≥ 85 cm for women.

### Settings and Study Subjects

Our study design was cross-sectional and the selection of respondents was voluntary and non-random. A total of 3,016 employees from two large petrochemical enterprises in Wuhan, Hubei Province (Central China) completed the physical examinations, laboratory tests, and face-to-face questionnaire surveys from October to December 2019. This study focused on the majority population aged 35–59 years old with high normal blood pressure, or IFG, or elevated blood cholesterol, or central obesity, or daily smoking (*n* =2,145). Respondents with any diagnosed NCDs, such as hypertension, diabetes, and hyperlipidemia, were excluded (*n* = 976). Furthermore, women who were trying to get pregnant, pregnant and breastfeeding were excluded due to that these populations had different diet recommendations in Chinese dietary guidelines 2016 (*n* = 124). Observations with abnormal body mass index (BMI <15 kg/m^2^ or BMI > 50 kg/m^2^) were pruned away (*n* = 29). In addition, observations with incomplete personal characteristics were censored (*n* = 195). Finally, 821 individuals were included in the present study.

### Data Collection

Data were collected by physical examinations, laboratory tests and structured questionnaires. Physical examination items included height, weight, waist circumference, SBP, and DBP. Laboratory test indexes included FPG and TC. Information of the high-risk population of NCDs on their demographic characteristics (gender, age, educational level, whether to smoke daily, physical activity) and the average daily food consumption (tubers, beans, cereals, fruits, vegetables, eggs, aquatic products, meat and poultry, nuts, milk and dairy production, oil and salt) in the last 6 months were recorded by trained interviewers through face-to-face interviews. The questionnaire was designed by the Chinese Center for Disease Control and Prevention. Hubei Provincial Center for Disease Control and Prevention was responsible for organization, training, implementation and quality control of the survey. The food frequency questionnaire (FFQ) we used was validated by comparing with data obtained by a totally 18-day 24-h recall throughout a year (21). The reproducibility of FFQ was evaluated at three levels between FFQ1 (conducted at the beginning of the year) and FFQ2 (conducted at the end of the year), i.e., comparison of the mean intake of foods; correlation analysis of their intake; and cross-classification and agreement on their corresponding intake. The results showed a high degree of reproducibility, and the correlation coefficients ranged from 0.43 to 0.90. Relative validity was tested by comparing the results of food consumption from both FFQ1 and FFQ2 with those from the average of the 18-day 24-h recall, and significant differences were revealed in most of foods. The crude correlation coefficient between FFQ1 and means of the 24-h recall ranged from 0.12 to 0.87, and between FFQ2 and means of the 24-h recall ranged from 0.33 to 0.85.

### Assessment of Food Consumption

We employed the CFP 2016 to evaluate dietary status of the high-risk population of NCDs. The CFP contains the recommended daily consumption quantity for five food groups (Figure 1): (1) cereals and beans; (2) fruits and vegetables; (3) animal products (eggs, aquatic products, meat and poultry); (4) soybeans and nuts, milk and its products; (5) oil and salt. Each of the 12 foods was individually assigned a specific value as a reference level. Both minimum and maximum consumption amount were set up for tubers, cereals, beans, fruits, vegetables, eggs, aquatic products, meat and poultry, nuts, and oil, which were defined as lower and upper bounds, respectively. Unlike this, only the minimum consumption level was proposed for milk and dairy production, while only the maximum consumption amount was recommended for salt. We thus defined these two values as the lower and upper bounds, respectively. When the real food consumption was lower than the lower bound of CFP 2016, it was defined as under-consumption; and when the real food consumption was higher than the upper bound, it was defined as over-consumption.

### Covariates

BMI was calculated by dividing the weight (kg) by the square of the height (m^2^) of each participant. According to the criteria recommended by Working Group on Obesity in China (22), it was further divided into four categorical levels: underweight (BMI < 18.5 kg/m^2^), normal weight (18.5 ≤ BMI < 24 kg/m^2^), overweight (24 ≤ BMI < 28 kg/m^2^), and obesity (BMI ≥28 kg/m^2^). Regular physical activity was defined as walking at least 6,000 steps per day.

### Statistical Analysis

Data was recorded into Excel 2016 and the SPSS 22 was used for statistical analysis. Descriptive statistical analyses on socio-demographic data and self-reported dietary intake data were reported as means, frequencies and percentages. After comparing self-reported dietary intakes with CFP's recommendations, we divided dietary patterns of each food group into three categories: under-consumption, meet-recommendation and over-consumption. Chi-square tests were conducted to compare the adherence to CFP of dietary intake among participants with different BMI and high-risk factors. One-way analysis of variance was used to compare differences in dietary intake among people with different high-risk factors of NCDs. All comparisons were two-tailed, and *p*-values < 0.05 were considered statistically significant.

## Results

### Sociodemographic Characteristics of Participants With Different High-Risk Factors of NCDs

Overall, 821 participants were included in the study, and 82.2% of them were males (Table 1). 42.1% of the participants aged 35–40 years old, followed by the participants aged 41–45 years old (33.4%). More than half (59.0%) of the participants had a high school education, and 190 (23.1%) had a college education or above. 58.6% of the participants were of normal weight, and 270 (32.9%) were overweight. 100 (12.2%) of the participants had three or more high-risk factors of NCDs, and 53.8% had only one high-risk factor of NCDs. Ninety-seven percent of the participants lacked the knowledge to walk 6,000 steps every day, and 64.7% were physical inactivity. Participants with high normal blood pressure accounted for 23.3%. The detection rate of IFG and elevated blood cholesterol were 3.5 and 49.6%, respectively. In addition, 31.4% of the participants were centrally obese. Males were more likely to have high normal blood pressure, central obesity and smoke daily. Participants with elevated blood cholesterol were more likely to be young adults and physical inactivity.

**Table 1:** Sociodemographic characteristics of participants with different high-risk factors of NCDs.

**Variables, ***N*** (%)**			**High-risk factors of NCDs**				
			**SBP: 130–139 mmHg, DBP: 85–89 mmHg**	**Smoking daily**	**6.1 mmol/L ≤FPG <7.0 mmol/L**	**5.2 mmol/L ≤TC <6.2 mmol/L**	**Waist circumference ≥90 cm for men and ≥85 cm for women**
Gender	Male	675 (82.2%)	168 (88.0%)*	441 (99.8%)**	23 (79.3%)	295 (72.5%)**	229 (88.8%)**
	Female	146 (17.8%)	23 (12.0%)	1 (0.2%)	6 (20.7%)	112 (27.5%)	29 (11.2%)
Age (years)	35~40	346 (42.1%)	89 (46.6%)	192 (43.4%)*	8 (27.6%)	148 (36.4%)**	111 (43.0%)
	41~45	274 (33.4%)	64 (33.5%)	133 (30.2%)	9 (31.0%)	133 (32.6%)	81 (31.4%)
	46~50	116 (14.1%)	23 (12.0%)	59 (13.3%)	5 (17.2%)	68 (16.7%)	39 (15.1%)
	51~59	85 (10.4%)	15 (7.9%)	58 (13.1%)	7 (24.2%)	58 (14.3%)	27 (10.5%)
Education level	Middle school or below	147 (17.9%)	46 (24.1%)*	78 (17.6%)*	5 (17.2%)	54 (13.3 %)**	55 (21.3%)*
	High school	484 (59.0%)	106 (55.5%)	280 (63.3%)	19 (65.5%)	234 (57.5%)	156 (60.5%)
	College or above	190 (23.1%)	39 (20.4)	84 (19.1%)	5 (17.3%)	119 (29.2%)	47 (1.2%)
BMI (kg/m^2^)	BMI <18.5	18 (2.2%)	3 (1.6%)*	8 (1.8%)	0 (0.0%)	10 (2.5%)*	1 (0.4%)**
	18.5 ≤ BMI <24	481 (58.6 %)	92 (48.2%)	263 (59.5%)	16 (55.2%)	266 (65.4%)	68 (26.4%)
	24 ≤ BMI <28	270 (32.9%)	80 (41.9%)	150 (33.9%)	10 (34.5%)	107 (26.3%)	140 (54.2%)
	BMI≥28	52 (6.3%)	16 (8.3%)	21 (4.8%)	3 (10.3%)	24 (5.8%)	49 (19.0%)
Number of high-risk	1	442 (53.8%)	57 (29.8%)**	151 (34.2%)**	7 (24.1%)**	162 (39.8%)**	71 (27.5%)**
factors of NCDs	2	279 (34.0%)	72 (37.7%)	204 (46.2%)	6 (20.7%)	170 (41.8%)	109 (42.2%)
	≥3	100 (12.2%)	62 (32.5%)	87 (19.6%)	16 (55.2%)	75 (18.4%)	78 (30.3%)
Whether to know that	Yes	25 (3%)	3 (1.6%)	12 (2.7%)	1 (3.4%)	20 (4.9%)*	7 (2.7%)
CFP recommends walking 6,000 steps every day	No	796 (97%)	188 (98.4%)	430 (97.3%)	28 (96.6%)	387 (95.1%)	251 (97.3%)
Regular physical	Yes	290 (35.3%)	60 (31.4%)	152 (34.4%)	9 (31.0%)	160 (39.3%)*	84 (32.6%)
activity	No	531 (64.7%)	131 (68.6%)	290 (65.6%)	20 (69.0%)	247 (60.7%)	174 (67.4%)

*NCDs, non-communicable diseases; BMI, body mass index; CFP, Chinese Food Pagoda 2016; SBP, Systolic Blood Pressure; DBP, Diastolic Blood Pressure; FPG, Fasting Plasma Glucose; TC, Total Cholesterol*.

* *P < 0.05*.

** *P < 0.001*.

### Dietary Intake of Participants Compared With CFP's Recommendation

Table 2 showed the real food consumption of the high-risk population of NCDs and the recommended food consumption in CFP. Most of the surveyed high-risk population of NCDs consumed less tubers, beans, fruits, eggs, nuts, milk and dairy products than the minimum amount recommended by CFP. Particularly, 99.1% of the participants' intake of milk and dairy products were less than the recommendation of CFP. 93.2% of the participants consumed less nuts than the CFP's recommendation. More than 80% of the participants consumed fewer beans and fruits than the CFP's recommendation. The average consumption of cereals, aquatic products, meat and poultry, oil and salt were more than the maximum amount recommended by CFP. The average intake of oil and salt were 48.3 g/d and 8.9 g/d, with 97.1% and 95.9% of the participants consuming excessive amounts, respectively. The average meat and poultry intake were 194.7 g/d, far exceeding the maximum recommended amount (40–75 g/d). The average consumption of vegetables was 306.9 g/d, meeting the recommendation of CFP (300–500 g/d). Males were more likely to consume excessive amounts of aquatic products and meat and poultry than females (*P* < 0.05).

**Table 2:** Dietary intake of participants compared with CFP's recommendation and the comparison between male and female.

**Food categories**	**Per capita intake (g/d)**	**CFP recommendation (g/d)**	**Under-consumption ***n*** (%)**	**Meet recommendation ***n*** (%)**	**Over-consumption ***n*** (%)**	**χ^2^ (Male vs. female)**	***P*** **-value**
Tubers	36.2	50–100	660 (80.4%)	98 (11.9%)	63 (7.7%)	5.959	0.051
Beans	20.8	50–150	745 (90.7%)	72 (8.8%)	4 (0.5%)	1.344	0.511
Cereals	431.3	250–400	60 (7.3%)	192 (23.4%)	569 (69.3%)	1.055	0.590
Fruits	104.6	200–350	668 (81.4 %)	133 (16.2%)	20 (2.4%)	5.963	0.051
Vegetables	306.9	300–500	503 (61.3%)	229 (27.9%)	89 (10.8%)	4.665	0.097
Eggs	34.7	40–50	483 (58.8%)	47 (5.7%)	291 (35.4%)	0.287	0.866
Aquatic product	87.2	40–75	292 (35.6%)	308 (37.5%)	221 (26.9%)	11.702	0.003
Meat and poultry	194.7	40–75	53 (6.5%)	109 (13.3%)	659 (80.3%)	8.216	0.016
Nuts	6.6	25–30	765 (93.2%)	24 (2.9%)	32 (3.9%)	1.198	0.549
Milk and dairy products	48.0	>300	814 (99.1%)	7 (0.9%)	0 (0.0%)	1.527	0.614
Oil	48.3	25–30	1 (0.1%)	23 (2.8%)	797 (97.1%)	5.347	0.069
Salt	8.9	<6	0 (0.0%)	34 (4.1%)	787 (95.9%)	3.436	0.068

*CFP, Chinese Food Pagoda 2016*.

### Comparison of the Dietary Intake Among Participants With Different BMI

Dietary patterns in the high-risk population of NCDs with different BMI were shown in Table 3. With the exception of cereals, oils, and salt, there were no significant differences in the proportion of adherence to CFP in other food categories among participants in different BMI groups. Participants with higher BMI were more likely to consume cereals over the recommendation of CFP (χ^2^ = 13.9, *P* < 0.05). Overweight and obese participants were more likely to consume oil (χ^2^ = 58.0, *P* < 0.05) and salt (χ^2^ = 9.0, *P* < 0.05) over the recommendation of CFP than those who were underweight or of normal weight.

**Table 3:** Comparison of the dietary intake among participants with different BMI.

**Food categories**	**Underweight**			**Normal**			**Overweight**			**Obesity**			**χ^2^**	***P***
	**A**	**B**	**C**	**A**	**B**	**C**	**A**	**B**	**C**	**A**	**B**	**C**		
Tubers	14 (77.8%)	3 (16.6%)	1 (5.6%)	388 (80.7%)	49 (10.2%)	44 (9.1%)	211 (78.1%)	41 (15.2%)	18 (6.7%)	47 (90.4%)	5 (9.6%)	0 (0.0%)	10.8	0.093
Beans	18 (100.0%)	0 (0.0%)	0 (0.0%)	430 (89.4%)	48 (10. 0%)	3 (0.6%)	250 (92.6%)	19 (7.0%)	1 (0.4%)	47 (90.4%)	5 (9.6%)	0 (0.0%)	4.3	0.634
Cereals	0 (0.0%)	6 (33.3%)	12 (66.7%)	35 (7.2%)	123 (25.6%)	323 (67.2%)	22 (8.1%)	45 (16.7%)	203 (75.2%)	3 (5.8%)	18 (34.6%)	31 (59.6%)	13.9	<0.05
Fruits	13 (72.2%)	5 (27.8%)	0 (0.0%)	391 (81.3%)	76 (15.8%)	14 (2.9%)	220 (81.4%)	45 (16.7%)	5 (1.9%)	44 (84.6%)	7 (13.5%)	1 (1.9%)	3.4	0.759
Vegetables	8 (44.4%)	6 (33.4%)	4 (22.2%)	294 (61.1%)	134 (27.9%)	53 (11.0%)	172 (63.7%)	74 (27.4%)	24 (8.9%)	29 (55.8%)	15 (28.8%)	8 (15.4%)	5.7	0.459
Eggs	12 (66.7%)	2 (11.1%)	4 (22.2%)	279 (58.0%)	28 (5.8%)	174 (36.2%)	158 (58.5%)	16 (5.9%)	96 (35.6%)	34 (65.4%)	1 (1.9%)	17 (32.7%)	4.0	0.683
Aquatic product	7 (38.9%)	6 (33.3%)	5 (27.8%)	170 (35.3%)	180 (37.5%)	131 (27.2%)	99 (36.7%)	97 (35.9%)	74 (27.4%)	16 (30.7%)	25 (48.1%)	11 (21.2%)	3.0	0.810
Meat and poultry	1 (5.6%)	3 (16.6%)	14 (77.8%)	35 (7.3%)	63 (13.1%)	383 (79.6%)	17 (6.3%)	36 (13.3%)	217 (80.4%)	0 (0.0%)	7 (13.5%)	45 (86.5%)	4.4	0.629
Nuts	16 (88.9%)	0 (0.0%)	2 (11.1%)	445 (92.5%)	17 (3.5%)	19 (4.0%)	253 (93.7%)	6 (2.2%)	11 (4.1%)	51 (98.1%)	1 (1.9%)	0 (0.0%)	6.4	0.377
Milk and dairy products	18 (100.0%)	0 (0.0%)	0 (0.0%)	475 (98.8%)	6 (1.2%)	0 (0.0%)	269 (99.6%)	1 (0.4%)	0 (0.0%)	52 (100.0%)	0 (0.0%)	0 (0.0%)	2.2	0.526
Oil	1 (5.6%)	0 (0.0%)	17 (94.4%)	0 (0.0%)	22 (4.6%)	459 (95.4%)	0 (0.0%)	1 (0.4%)	269 (99.6%)	0 (0.0%)	0 (0.0%)	52 (100.0%)	58.0	<0.05
Salt	0 (0.0%)	2 (11.1%)	16 (88.9%)	0 (0.0%)	26 (5.4%)	455 (94.6%)	0 (0.0%)	4 (1.5%)	266 (98.5%)	0 (0.0%)	2 (3.8%)	50 (96.2%)	9.0	<0.05

*Values are presented as number and percentage [n (%)]. BMI, Body Mass Index; underweight, BMI ≤ 18.5 kg/m^2^; normal weight, 18.5 ≤ BMI <24 kg/m^2^; overweight, BMI 24 ≤ BMI <28 kg/m^2^; obesity, BMI ≥ 28 kg/m^2^; A, the number and percentage of study participants whose food intakes are below the recommendation; B, the number and percentage of study participants whose food intakes are with the recommendation; C, the number and percentage of study participants whose food intakes are above the recommendation. For each food item, the P-value was related to the comparison on the proportion of participants with different dietary intakes across the four body mass index groups*.

### Comparison of the Dietary Intake Among Participants With Different High-Risk Factors

Table 4 showed the dietary intake of participants with different high-risk factors of developing NCDs. There were significant differences in average intake of cereals among participants with different high-risk factors (*F* = 7.1, *P* < 0.001). Participants with IFG had the highest consumption of cereals (459.9 g/d), whereas people with elevated blood cholesterol had the lowest consumption (413.5 g/d). Daily smokers consumed more beans than participants with the other four high-risk factors (*F* = 3.6, *P* < 0.05). The fruit intake of those with elevated blood cholesterol (118.3 g/d) was the highest, followed by those with IFG (100.9 g/d) (*P* < 0.05). The consumption of meat and poultry (214.9 g/d), and aquatic products (104.8 g/d) in the central obesity group were significantly higher than those in the other groups (*P* < 0.05). The lowest egg intake (31.8 g/d) and the highest milk and dairy products intake (56.7 g/d) were found in participants with elevated blood cholesterol (*P* < 0.05).

**Table 4:** Comparison of the dietary intake among participants with different high-risk factors.

**Food categories (g/d)**	**High-risk factors of NCDs**					**F**	***P***
	**SBP: 130–139 mmHg, DBP: 85–89 mmHg**	**Smoking daily**	**6.1 mmol/L ≤FPG <7.0 mmol/L**	**5.2 mmol/L ≤TC <6.2 mmol/L**	**waist circumference ≥90 cm for men and ≥85 cm for women**		
Tubers	38.9	37.0	30.9	30.3	35.6	4.2	<0.05
Beans	16.3	24.2	13.6	21.5	16.6	3.6	<0.05
Cereals	454.4	455.7	459.9	413.5	442.8	7.1	<0.001
Fruits	92.0	96.7	100.9	118.3	99.3	4.1	<0.05
Vegetables	301.1	305.4	346.4	312.3	291.0	1.1	0.375
Eggs	38.4	35.3	35.8	31.8	35.0	2.7	<0.05
Aquatic product	79.9	88.8	80.1	69.7	104.8	3.6	<0.05
Meat and poultry	205.9	196.4	158.2	165.4	214.9	4.3	<0.05
Nuts	6.0	7.2	5.2	6.6	6.8	0.5	0.733
Milk and dairy products	43.7	42.1	43.8	56.7	43.1	3.5	<0.05
Oil	48.2	48.1	50.4	48.8	48.5	0.7	0.599
Salt	9.0	8.9	9.3	9.1	8.9	0.8	0.519

*NCDs, non-communicable diseases; SBP, Systolic Blood Pressure; DBP, Diastolic Blood Pressure; FPG, Fasting Plasma Glucose; TC, Total Cholesterol*.

### Dietary Intake of Participants With Different High-Risk Factors Compared With CFP's Recommendation

**Figure 2:**
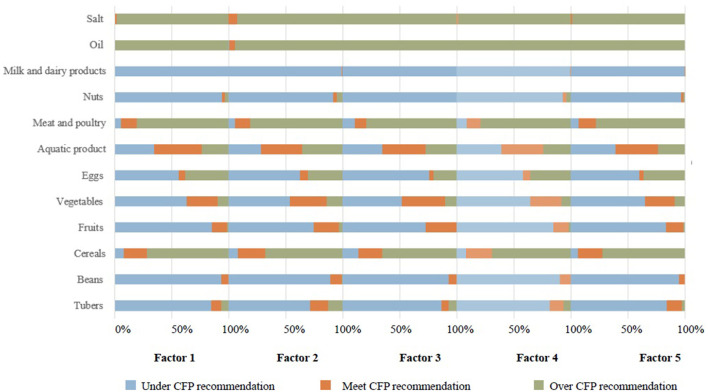
Dietary intake among participants with different high-risk factors compared to the CFP's recommendation. CFP, Chinese Food Pagoda 2016; SBP, Systolic Blood Pressure; DBP, Diastolic Blood Pressure; FPG, Fasting Plasma Glucose; TC, Total Cholesterol; Factor 1, SBP, 130–139, DBP 85–89 mmHg; Factor 2, Smoking; Factor 3, 6.1 mmol/L ≤ FPG< 7.0 mmol/L; Factor 4, 5.2 mmol/L ≤ TC< 6.2 mmol/L; Factor 5, waist circumference ≥90 cm for men and ≥85 cm for women.

We made a comparison to explore whether there was a discrepancy in food adherence to CFP between participants with a high-risk factor and those without such a high-risk factor (Supplementary Table 1). The intake of oil and salt of the participants with high normal blood pressure were more likely to exceed the maximum recommended amounts of CFP (*P* < 0.05). More than 80% of the participants with high normal blood pressure consumed less tubers, beans, fruits, nuts, milk and dairy products than CFP's recommendation (Figure 2). Daily smokers were less likely to consume tubers, eggs, fruits, and vegetables (*P* < 0.05), and were more likely to consume aquatic products, oil and salt (*P* < 0.05). 81.0% of the smokers ate more meat and poultry than the CFP's recommendation. 98.9% of the smokers consumed less milk and dairy products than the CFP's recommendation. Most people with IFG had a higher intake of cereals, meat and poultry, oil and salt than the CFP's recommendation. Participants with elevated blood cholesterol consumed more meat and poultry, oil and salt than CFP's recommendation (*P* < 0.05). The intake of tubers, beans, nuts, milk and poultry among central obese people were significantly less than the CFP's recommendation (*P* < 0.05), but the intake of oil was higher than CFP's recommendation (*P* < 0.05).

## Discussion

NCDs is a critical public health issue in China, and the prevalence was 596.65 per 100,000 (19). Balanced diet is one of the behaviors that can prevent and reduce the severity of NCDs (23). Our findings showed that the average consumption of cereal, aquatic products, meat and poultry, oil, and salt among people with high-risk factors of NCDs were significantly higher than the upper bound of CFP's recommendation. To the contrary, the average consumption of tubers, beans, fruits, eggs, nuts, as well as milk and dairy products were significantly less than the lower bound of CFP's recommendation. The food group whose average consumption fell into the recommended boundary was vegetables. There were no significant gender differences in dietary intake compared with CFP's recommendation, except for aquatic products and meat and poultry. This result may be related to men's preference for higher-energy aquatic products and meat and poultry, which are more convenient to provide more energy and meet their physical demands.

Studies have shown that over-consumption of oil, and meat and poultry would lead to the increased risk of NCDs (24, 25), which is consistent with the results of our study. Palm oil is one of the most commonly used vegetable oils and is found in around half of frequently used foods such as cooking oil, shortening and margarine (26). Polyunsaturated fats from vegetable oils have important cardiovascular benefits, but the excessive consumption of saturated fat in palm oil would contribute to obesity (27) and increase blood levels of atherogenic low-density lipoprotein cholesterol (28). A meta-analysis found a significant relationship of increased palm oil consumption with higher mortality from ischemic heart disease (29). Therefore, this is one of the limitations of CFP 2016 which only provided a rather crude dietary guideline using broad food categories, such as oil. It is advised to avoid excessive intake of oils that are high in saturated fat in the high-risk population of NCDs.

Nutrition transition is rapidly changing the types and amount of fat used in China. In our study, excessive consumption of meat and poultry was common in the high-risk population of NCDs, especially in those with central obesity. Furthermore, Chinese people tend to eat more meat and poultry in winter, particularly during spring festival, which would lead to an underestimate of meat and poultry intake among people at high risk of NCDs. It is suggested to steam or poach the meat to retain more nutrients and less fat. Epidemiologic evidence has shown that diets containing substantial amount of meat and poultry probably increase the risk of coronary heart disease and some cancers (30). In India, there were positive associations of the animal food pattern with cardio-metabolic risk factors (31). In Australia, over-consumption of meat and poultry was associated with higher BP and central obesity (32). Therefore, it is urgent to reduce the intake of meat and poultry for people at high risk of NCDs, especially for men. Meanwhile, soybean and soy products are encouraged as an alternative of meat because their taste was similar to meat and were commonly consumed in the Chinese diet (33).

The findings indicated that milk and dairy products were under-consumed in the high-risk population of NCDs. Milk and dairy products contain multiple nutrients including protein, calcium, magnesium, phosphorus, potassium, zinc, selenium, vitamin A, riboflavin, vitamin B-12, and pantothenic acid. Many of the beneficial effects of milk and dairy products might be due to the interactions between nutrients, not just the individual effects of these nutrients. It is suggested that the consumption of dairy products, whether low-fat or high-fat, may ameliorate characteristics of the metabolic syndrome and markedly decrease the risk of diabetes and cardiovascular diseases (34–36). Risk factors such as insulin resistance, increased blood pressure, dyslipidemia, and abdominal obesity would be improved with adequate intake of protein and dairy products. Therefore, it is essential for the high-risk population of NCDs to consume milk and dairy products in accordance with the recommended amount of CFP.

Participants with IFG had the highest average intake of cereals compared to those with other high-risk factors of developing NCDs. People who regularly consumed highly processed cereals are prone to rapid rises and falls in blood glucose, resulting in a high glycemic load. Individuals with underlying insulin resistance especially those who were overweight were more susceptible to the adverse metabolic effects of high glycemic diets, which was in line with the results of a prospective study in Shanghai (37). Furthermore, a previous study showed that the over-intake of cereals was positively associated with overweight (14). As the prevalence of overweight and obesity in China continues to increase and the population becomes more sedentary, the role of refined carbohydrate intake in the development of diabetes and other metabolic diseases will become more apparent in the coming years.

Data of subjects with high normal blood pressure from clinical trials showed that 40% developed hypertension over 2 years and 63% over four years (38). It was estimated that about two-thirds of the burden of cerebrovascular diseases and one half of the burden of coronary diseases were attributed to non-optimal blood pressure levels. The World Health Report 2002 identified lowering blood pressure by reducing dietary salt as a potentially cost-effective means of preventing cardiovascular disease (39). In China, salt has been widely used in many traditional diets and salt intake in the high-risk population of NCDs seriously exceeded the recommended standard. These findings suggest that a salt substitute could be a low-cost alternative or adjunct to drug therapy for people who habitually consume high amounts of salt (40), which is particularly attractive for primary prevention of hypertension in large populations.

Over the past few decades, the diet of many Chinese people had shifted from coarser grain and nutrient-rich legumes to refined grains, animal-source foods, edible oils and snack foods (41). Meanwhile, they have reduced physical movement and increased the sitting time, which increased the prevalence of overweight and obesity (42). The CFP recommended to keep a regular physical activity which amounted to at least 6,000 steps each day (43). In our study, most of the participants lacked the knowledge to walk 6,000 steps every day, and nearly half of people at high risk of NCDs took <6,000 steps daily. Avoiding ingesting excessive food and physical inactivity is the best way to maintain energy balance. It is particularly important to strengthen guidance on balanced diet and physical activity for people at high risk of NCDs. Several limitations should be mentioned in the present study. Firstly, the subjects in our study were from two large petrochemical enterprises, and further studies with national representativeness are warranted to test this conclusion. Thus, our results should be interpreted with caution. Secondly, the impact of different food categories on various high-risk factors of NCDs requires further clinical research. Thirdly, we collected data of dietary intake through self-reporting, which may contribute to recall bias. Fourthly, we did not take sugar into account in this study because the guidelines for sugar in Chinese Dietary Guidelines 2016 are not presented in the CFP.

In conclusion, our study indicated that the dietary status of Chinese adults at high-risk of NCDs was far away from the standards noted in CFP 2016. A diet pattern with more fruit, milk and dairy products, but less meat and poultry, oil and salt, should be promoted in the high-risk population of NCDs, which might address modifiable risk factors and thus reduce the burden of NCDs in China. Future research should focus on regularly and systematically assessing the adherence to nutritional guidelines in the high-risk population of NCDs, providing evidence for dietary behavior interventions and health policy formulation.

## Data Availability Statement

The original contributions presented in the study are included in the article/Supplementary Material, further inquiries can be directed to the corresponding authors.

## Ethics Statement

This study was conducted according to the guidelines laid down in the Declaration of Helsinki. All procedures involving study participants were approved by the Research Ethics Committee in Tongji Medical College, Huazhong University of Science and Technology, Wuhan, China. Written informed consent was obtained from all participants before the investigation.

## Author Contributions

QJ and SC: conceptualization. QJ, QY, and SC: methodology. YL and SW: formal analysis and writing—review and editing. QJ and QY: writing—original draft preparation. SC: supervision and funding acquisition. All authors have read and agreed to the published version of the manuscript.

## Funding

This work was supported by the Fundamental Research Funds for the Central Universities, Huazhong University of Science and Technology (2020WKZDJC015). The funders had no role in the design of the study; in the collection, analyses, or interpretation of data; in the writing of the manuscript, or in the decision to publish the results.

## Conflict of Interest

The authors declare that the research was conducted in the absence of any commercial or financial relationships that could be construed as a potential conflict of interest.

## Publisher's Note

All claims expressed in this article are solely those of the authors and do not necessarily represent those of their affiliated organizations, or those of the publisher, the editors and the reviewers. Any product that may be evaluated in this article, or claim that may be made by its manufacturer, is not guaranteed or endorsed by the publisher.
