# Supplementary material for: Adherence to the Chinese Food Pagoda in the High-Risk Population of Non-communicable Diseases Aged 35–59 in Central China
Source: Front Nutr. 2022 Mar 4;9:781963. doi: 10.3389/fnut.2022.781963 (PMC8931698; doi:10.3389/fnut.2022.781963)
Supplement: Supplementary file 1 [file Table_1.DOCX]

Appendix Table 1. Dietary intake of participants with different high-risk factors compared with CFP’s recommendation.

| **Food group** | **Under-consumption**  **（%）** | **Meet recommendation**  **（%）** | **Over-consumption**  **（%）** | **χ^2^** | **P** |
| --- | --- | --- | --- | --- | --- |
| **SBP: 130-139, DBP: 85-89mmHg** | | | | | |
| Tubers | 84.8 | 8.9 | 6.3 | 3.2 | 0.206 |
| Beans | 93.2 | 6.8 | 0.0 | 2.5 | 0.291 |
| Cereals | 7.9 | 20.4 | 71.7 | 1.2 | 0.536 |
| Fruits | 85.3 | 13.1 | 1.6 | 2.7 | 0.255 |
| Vegetables | 62.8 | 27.3 | 9.9 | 0.3 | 0.851 |
| Eggs | 56.0 | 5.8 | 38.2 | 0.9 | 0.646 |
| Aquatic product | 34.6 | 41.8 | 23.6 | 2.4 | 0.304 |
| Meat and poultry | 5.2 | 14.2 | 80.6 | 0.7 | 0.699 |
| Nuts | 94.2 | 2.1 | 3.7 | 0.7 | 0.723 |
| Milk and dairy products | 99.5 | 0.5 | 0.0 | 0.3 | 1.000 |
| Oil | 0.0 | 0.0 | 100.0 | 7.5 | 0.024 |
| Salt | 0.0 | 1.6 | 98.4 | 4.1 | 0.039 |
| **Smoking daily** |  |  |  |  |  |
| Tubers | 71.0 | 16.1 | 12.9 | 58.1 | 0.000 |
| Beans | 89.4 | 10.0 | 0.6 | 2.5 | 0.293 |
| Cereals | 7.9 | 24.2 | 67.9 | 1.0 | 0.592 |
| Fruits | 74.2 | 22.6 | 3.2 | 32.5 | 0.000 |
| Vegetables | 53.6 | 31.9 | 14.5 | 26.3 | 0.000 |
| Eggs | 62.2 | 7.0 | 30.8 | 10.6 | 0.005 |
| Aquatic product | 28.3 | 36.2 | 35.5 | 41.1 | 0.000 |
| Meat and poultry | 5.2 | 13.8 | 81.0 | 2.6 | 0.274 |
| Nuts | 91.6 | 3.2 | 5.2 | 4.6 | 0.099 |
| Milk and dairy products | 98.9 | 1.1 | 0.0 | 0.9 | 0.461 |
| Oil | 0.2 | 5.2 | 94.6 | 21.2 | 0.000 |
| Salt | 0.0 | 7.2 | 92.8 | 23.2 | 0.000 |
| **6.1 mmol/L ≤**[**FBG**](javascript:;)**< 7.0 mmol/L** | | | | | |
| Tubers | 86.2 | 6.9 | 6.9 | 0.8 | 0.674 |
| Beans | 93.1 | 6.9 | 0.0 | 0.3 | 0.867 |
| Cereals | 13.8 | 20.7 | 65.5 | 1.9 | 0.390 |
| Fruits | 72.4 | 27.6 | 0.0 | 3.4 | 0.180 |
| Vegetables | 51.7 | 37.9 | 10.4 | 1.5 | 0.463 |
| Eggs | 75.9 | 3.4 | 20.7 | 3.6 | 0.165 |
| Aquatic product | 34.5 | 37.9 | 27.6 | 0.1 | 0.992 |
| Meat and poultry | 10.3 | 10.4 | 79.3 | 0.9 | 0.637 |
| Nuts | 100.0 | 0.0 | 0.0 | 2.2 | 0.333 |
| Milk and dairy products | 100.0 | 0.0 | 0.0 | 0.3 | 1.000 |
| Oil | 0.0 | 0.0 | 100.0 | 0.9 | 0.636 |
| Salt | 0.0 | 0.0 | 100.0 | 1.3 | 0.627 |
| **5.2 mmol/L ≤TC< 6.2 mmol/L** | | | | | |
| Tubers | 81.1 | 12.3 | 6.6 | 1.3 | 0.531 |
| Beans | 90.4 | 9.4 | 0.5 | 0.1 | 0.949 |
| Cereals | 7.9 | 23.1 | 69.0 | 0.4 | 0.828 |
| Fruits | 84.5 | 13.3 | 2.2 | 5.4 | 0.066 |
| Vegetables | 64.4 | 27.0 | 8.6 | 5.2 | 0.073 |
| Eggs | 58.2 | 5.9 | 35.9 | 0.1 | 0.936 |
| Aquatic product | 38.8 | 36.9 | 24.3 | 4.5 | 0.105 |
| Meat and poultry | 8.6 | 12.3 | 79.1 | 6.5 | 0.039 |
| Nuts | 93.1 | 2.7 | 4.2 | 0.3 | 0.862 |
| Milk and dairy products | 99.0 | 1.0 | 0.0 | 0.2 | 0.723 |
| Oil | 0.0 | 0.0 | 100.0 | 24.3 | 0.000 |
| Salt | 0.0 | 1.2 | 98.8 | 17.3 | 0.000 |
| **Waist circumference: male ≥90cm, female ≥85cm** | | | | | |
| Tubers | 83.7 | 13.6 | 2.7 | 13.4 | 0.001 |
| Beans | 94.6 | 5.4 | 0.0 | 7.2 | 0.027 |
| Cereals | 6.2 | 21.7 | 72.1 | 1.5 | 0.471 |
| Fruits | 83.3 | 15.1 | 1.6 | 1.7 | 0.435 |
| Vegetables | 65.1 | 25.6 | 9.3 | 2.5 | 0.294 |
| Eggs | 59.7 | 3.9 | 36.4 | 2.4 | 0.302 |
| Aquatic product | 39.1 | 37.3 | 23.6 | 2.9 | 0.239 |
| Meat and poultry | 6.6 | 15.1 | 78.3 | 1.2 | 0.562 |
| Nuts | 96.5 | 2.3 | 1.2 | 8.1 | 0.017 |
| Milk and dairy products | 99.6 | 0.4 | 0.0 | 1.0 | 0.443 |
| Oil | 0.0 | 0.0 | 100.0 | 11.3 | 0.003 |
| Salt | 0.0 | 1.2 | 98.8 | 8.4 | 0.002 |
